# Petrophysical Properties (Density and Magnetization) of Rocks from the Suhbaatar-Ulaanbaatar-Dalandzadgad Geophysical Profile in Mongolia and Their Implications

**DOI:** 10.1155/2013/791918

**Published:** 2013-11-07

**Authors:** Tao Yang, Jintian Gao, Zuowen Gu, Baatarkhuu Dagva, Batsaikhan Tserenpil

**Affiliations:** ^1^Institute of Geophysics, China Earthquake Administration, Beijing 100081, China; ^2^Research Center of Astronomy and Geophysics, Mongolian Academy of Sciences, 210351 Ulaanbaatar, Mongolia

## Abstract

Petrophysical properties of 585 rock samples from the Suhbaatar-Ulaanbaatar-Dalandzadgad geophysical profile in Mongolia are presented. Based on the rock classifications and tectonic units, petrophysical parameters (bulk density, magnetic susceptibility, intensity of natural remanent magnetization, and Köenigsberger ratio) of these rocks are summarized. Results indicate that (1) significant density contrast of different rocks would result in variable gravity anomalies along the profile; (2) magnetic susceptibility and natural remanent magnetization of all rocks are variable, covering 5-6 orders of magnitude, which would make a variable induced magnetization and further links to complex magnetic anomalies in ground surface; (3) the distribution of rocks with different lithologies controls the pattern of lithospheric magnetic anomaly along the profile. The petrophysical database thus provides not only one of the keys to understand the geological history and structure of the profile, but also essential information for analysis and interpretation of the geophysical (e.g., magnetic and gravity) survey data.

## 1. Introduction

Spatial and temporal variations in petrophysical property record the evolution process of the earth crust [1]. Consequently, knowledge of petrophysical properties is not only important basic data for regional geological and geophysical studies, but also essential for detailed potential field interpretations and modeling of source structures [2–4]. Measurements and analysis of petrophysical property are thus crucial for physically understanding the petrology and calibrating geophysical parameters [5, 6].

Mongolian plateau is part of the Central Asian Orogenic Belt (CAOB), one of the largest orogens on the Earth, and considered to have evolved over some 800 Ma, from the latest Mesoproterozoic to the late Palaeozoic [7–9]. The formation and tectonic evolution of the CAOB followed contraction and closure of the Paleo-Pacific, Paleo-Asian, Paleo-Tethyan, and the Tethyan oceans, respectively [10]. In the eastern Asian continent, Mesozoic and Cenozoic circum-Pacific orogens, which originated from subduction of the Pacific Plate under the Asian continent, are superimposed on the various abovementioned blocks and orogens. In addition, Tianshan-Mongolia-Lake Baikal seismic belt situated in the interior of continental Asia is one of the most seismically active regions in the world. Many earthquakes with Ms > 7 have occurred in it during the twentieth century [11]. Consequently, geophysical observations and studies in Mongolia are not only important for understanding the crust material components and structure of the CAOB, but also crucial for understanding the deep structure environment and mechanism of the strong earthquakes occurred in this seismic belt.

**Figure 1:**
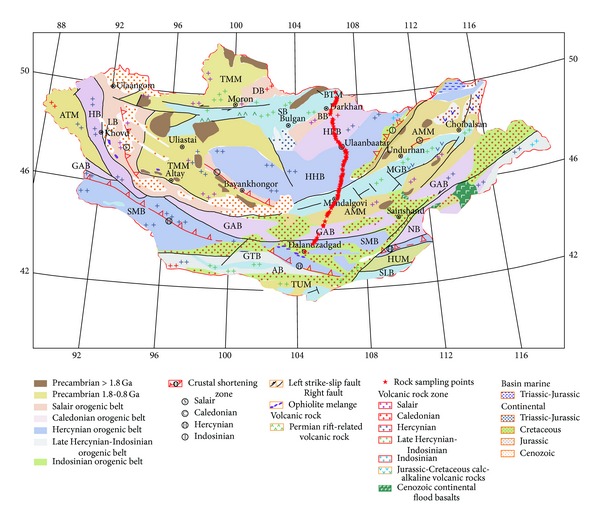
Outline of tectonic map of Mongolia with the sampling points of rock samples along the Suhbaatar-Ulaanbaatar-Dalandzadgad geophysical profile (compiled after Wang et al. [13]). *Tectonic Units*. North Mongolia: Tuva-Mongolian Massif (TMM); Argun-Mongolian Massif (AMM); Altai-Mongolian Massif (ATM); Buteel Massif (BTM); Lake belt (LB); Bayangol belt (BB); Dzhida belt (DB); Haraa belt (HRB); Gobi-Altai-Mandalgovi belt (GAB); Hangai-Hentii belt (HHB); Selenge belt (SB); Middle Gobi belt (MGB). South Mongolia: Tsagaan Uul Massif (TUM); Hutag Uul Massif (HUM); Nuhetdavaa belt (NB); Gobi-Tienshan belt (GTB); South Mongol belt (SMB); Atas Bogd belt (AB); Sulinheer belt (SLB).

One of the most important bases of interpretation and analysis of the crust material components and structure based on geophysical data is the petrophysical properties of rocks [12]. Up to date, however, few geophysical surveys have been carried out in Mongolia, and scarce petrophysical data of rocks from Mongolia have been documented. In 2011, an integrated magnetic and gravity survey was conducted along the profile from Suhbaatar to Dalandzadgad via Ulaanbaatar (Figure 1), which is the first long (~800 km) geophysical profile conducted in Mongolia. Meanwhile, 585 hand samples were collected from outcrops along the profile. In laboratory, petrophysical properties, including bulk density, magnetic susceptibility, and intensity of natural remanent magnetization, were measured. In this paper, these petrophysical parameters were analyzed statistically according to lithology and tectonic units, to discuss their implications and to provide constraints on the magnetic and density parameters for inversion of magnetic and gravity data.

## 2. Geological Backgrounds

Mongolia occupies the heart of the CAOB and an interior portion of the Eurasian Plate [15]. Geologically, it is an important link between the Siberian craton, essentially an amalgamation of lower Paleozoic terranes, and northern China, an area of complex middle Paleozoic-Tertiary suturing and tectonics [16]. The whole territory is cut by several near EW-trending arc faults and some NW- and NE-trending faults. Among them, two major EW-trending fault zones (the Hangay-Orhon Gol fault zone in the north and the Khanbogd-Undurshil fault zones in the south) divide the territory of Mongolia into three major tectonic domains: the northern part is the Baikal fold system (i.e., Tuva-Mongol Massif), the middle one is Caledonian fold system, and the Hercynia fold system is located in the south [13, 16]. Geological maps reveal the variety and complexity of rock types and structures, with representatives of all geological ages from Precambrian to Quaternary [17]. The rocks record successive episodes of terrane accretions and consequent deformation.

## 3. Samples and Methods

During the fieldwork in 2011, 585 hand samples were collected from 117 sampling points at outcrops along the Suhbaatar-Ulaanbaatar-Dalandzadgad geophysical profile (Figure 1). The natural cuts and/or road cuts were preferred as the best outcrops. At each sampling point, the outer surface (typically ca. 5 cm) was removed prior to sampling, to reduce the weathering effects; 5 hand samples (each one is about 5 cm × 5 cm × 5 cm) were collected to represent common rock types. In total, 235 sedimentary rock samples, 45 metamorphic rock samples, and 305 igneous rock samples were obtained.

In laboratory, all samples were cut into standard-size cubes or cylinders. Magnetic susceptibility (*κ*) was measured using an AGICO MFK1-FA Kappabridge magnetic susceptibility meter at a frequency of 976 Hz and a field intensity of 200 A/m. Intensity of natural remanent magnetization (NRM) was measured using a Minispin spinner magnetometer. Köenigsberger ratio (*Q*) is defined as the ratio of remanent to induced magnetization in a standard geomagnetic field of 5 × 10^4^ nT, to measure the relative importance of induced and remanent magnetization. Bulk density (*ρ*) was measured based on the principle of the Archimedes method [18], which involved weighing samples in air and when immersed in distilled water.

## 4. Basic Petrophysical Properties

**Figure 2:**
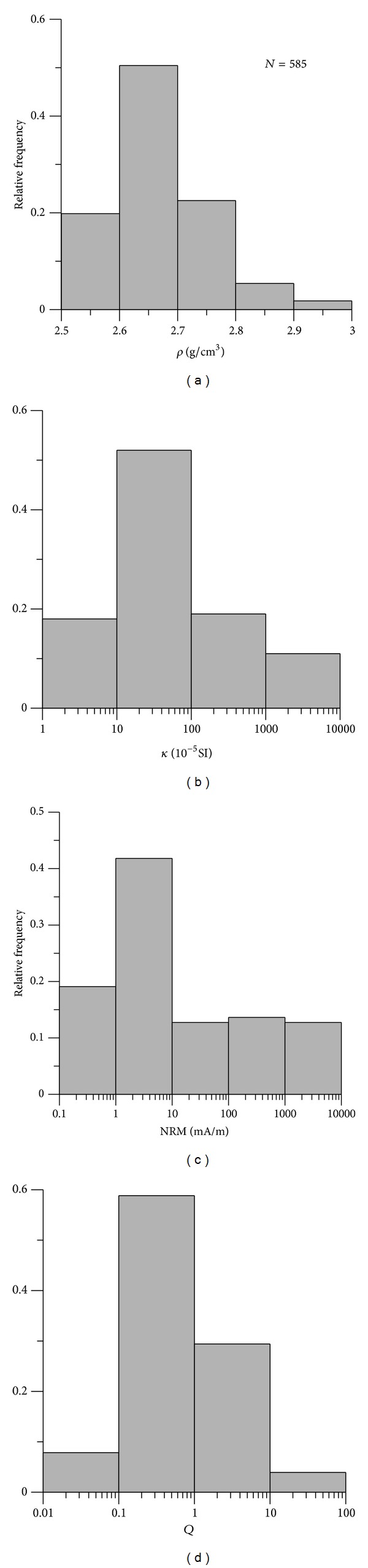
Histogram for (a) density (*ρ*), (b) magnetic susceptibility (*κ*), (c) natural remanent magnetization (NRM), and (d) Köenigsberger ratio (*Q*) of all rock samples from the Suhbaatar-Ulaanbaatar-Dalandzadgad geophysical profile in Mongolia.

The general aspects of the petrophysical properties are displayed in the plots of the total samples (Figure 2). Generally, density of a rock is the sum of the products of the mineral densities and the mineral volume contents [19]. *ρ* shows a unimodal distribution with dominant values ranging 2.6~2.8 g/cm^3^ (Figure 2(a)). *κ* and NRM are variable, covering 5~6 orders of magnitude (Figures 2(b) and 2(c)). It is clearly seen that most rocks have low *Q* values (i.e., induced magnetization dominates) (Figure 2(d)).

In the following, petrophysical data for three major lithologies are presented.

### 4.1. Sedimentary Rocks

Density of sedimentary rocks depends on the density of their compositional minerals, porosity, and the density of filling liquid and gas [19]. Generally, average *ρ* value of chemical sedimentary rocks (dolostone, limestone, and siliceous rocks) is higher than that of clastic sedimentary rocks (mudstone, and siltstone, sandstone). For chemical sedimentary rocks, average *ρ* value of carbonate rocks (limestone and dolostone) is higher than that of siliceous rocks. In clastic rocks, mudstone has the highest *ρ* value, whereas average *ρ* value of siltstone is the lowest (Table 1).

**Figure 3:**
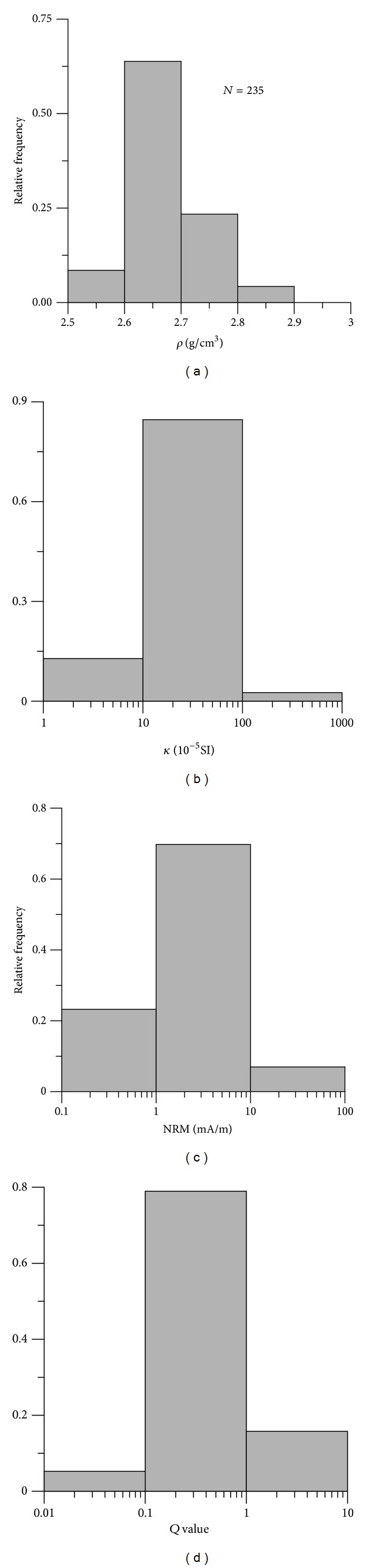
Histogram for (a) *ρ*, (b) *κ*, (c) NRM, and (d) *Q* of sedimentary rock samples.

Magnetic susceptibility of sedimentary rock mainly depends on the composition and content of accessory minerals (e.g., magnetite, maghemite, hematite, and iron hydroxides) [19]. *κ* and NRM of sedimentary rock mainly range in 10~100 × 10^−5^ SI and 1~10 mA/m, respectively (Figures 3(b) and 3(c)). Among them, clastic sedimentary rocks have higher *κ* and NRM than chemical sedimentary rocks (Table 1). Generally, *Q* value is lower than 1 (Figure 3(d)), indicating that their magnetization is dominated by induced magnetization.

### 4.2. Igneous Rocks

**Figure 4:**
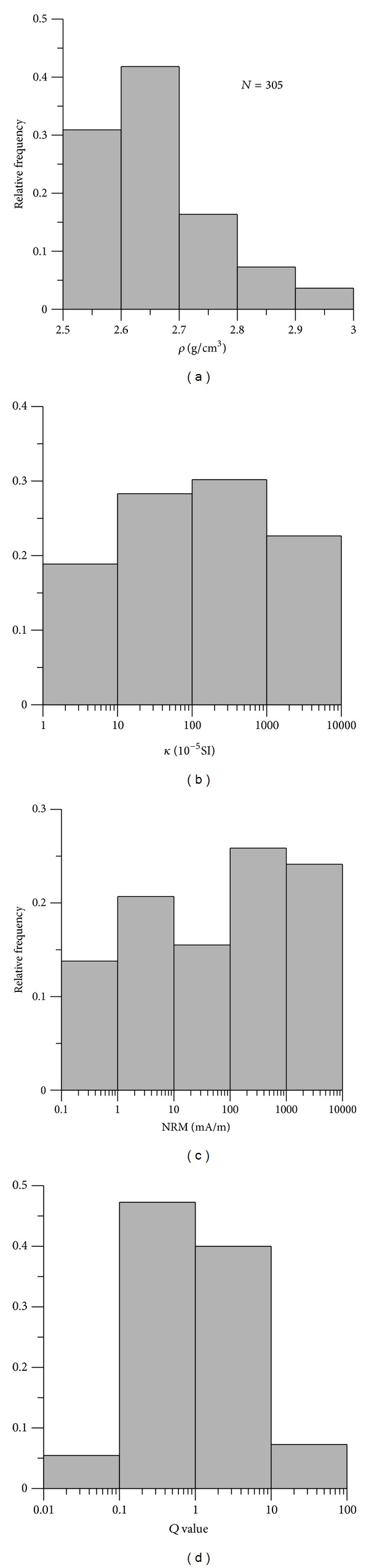
Histogram for (a) *ρ*, (b) *κ*, (c) NRM, and (d) *Q* of igneous rock samples.

**Figure 5:**
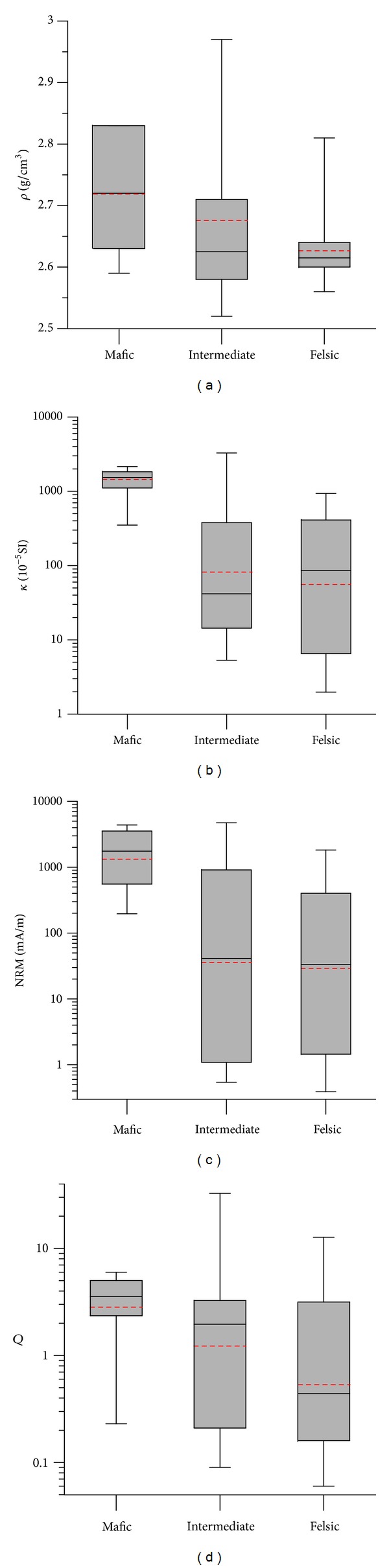
Box plot for (a) *ρ*, (b) *κ*, (c) NRM, and (d) *Q* of basic, intermediate, and felsic igneous rocks. Red dash lines indicate the average values of the corresponding petrophysical parameters.

Density of igneous rocks depends almost exclusively on the mineralogical and chemical composition of these rocks, it increases with the increasing content of dark minerals [19]. Igneous rocks have a wide range of *ρ* distribution, with dominant values of 2.5~2.8 g/cm^3^ (Figure 4(a)). Extrusive rocks (e.g., basalt and dacite) have much higher *ρ* than those of intrusive rocks; among them, *ρ* of plutonic rock is generally higher than that of hypabyssal rock (Table 2). The mafic basalt has the highest *ρ*, with an average value of ~2.72 g/cm^3^, followed by those of intermediate igneous rocks (e.g., tuff, diorite, syenite, and dacite) (mean *ρ*~2.67 g/cm^3^), and the felsic granite and porphyry generally have the lowest *ρ*, with average value of ~2.63 g/cm^3^ (Table 3 and Figure 5(a)). Geological age also has a considerable influence on density of rock. Rocks with the same lithology may display different densities, deviating by 5~20% from the average value, at different ages. For example, average *ρ* value of felsic granite increases with the decreasing age, Ordovician (2.62 g/cm^3^) → Devonian (2.65 g/cm^3^) → Permian (2.73 g/cm^3^) → Triassic (2.76 g/cm^3^).

*κ* and NRM of igneous rock are variable (Figures 4(b) and 4(c)) and increase significantly with the decreasing content of silica. Namely, felsic rocks have the lowest *κ* and NRM values, followed by intermediate rocks, and mafic rocks show the highest *κ* and NRM values (Table 3 and Figures 5(b) and 5(c)). For example, average *κ* value for felsic granite and intermediate dacite is about ~80 and ~350 × 10^−5^ SI, respectively. *κ* for mafic basalt ranges from hundreds to thousands 10^−5^ SI, with an average value of 1300 × 10^−5^ SI (Table 3). Mafic rocks have considerable NRM, ranging between hundreds and thousands mA/m, which often makes an important contribution to the total magnetization (Table 3). In addition, it is found that extrusive rocks (e.g., basalt and dacite) have much higher *κ* compared to plutonic rocks; hypabyssal and pyroclastic rocks have relatively lower *κ* (Table 2).

Generally, samples with *Q* > 1 are comparable with those having *Q* < 1 (Figure 4(d)). Among them, *Q* value of mafic rocks ranges between 2 and 6, indicating the dominance of natural remanent magnetization with respect to the induced magnetization (Table 2). NRM of intermediate rocks is comparable with their induced magnetization; in contrast, felsic plutonic rocks have much lower *Q* values (generally <1), indicating the strong contribution of induced magnetization to the total magnetization. In general, extrusive and pyroclastic rocks that formed on earth's surface have much higher *Q* values; hypabyssal rocks that formed near the surface have moderate *Q* values, whereas the plutonic rocks have the lowest *Q* values (Table 2).

### 4.3. Metamorphic Rocks

Density of metamorphic rocks depends not only on the mineralogical composition of the parent rock, but also on the degree of metamorphism and diagenesis [19]. Few metamorphic rocks are present along the profile, which are dominated by regional metamorphic rocks. Among them, marble has the highest *ρ*, followed by gneiss, which is denser than quartzite (Table 4). Magnetic susceptibility of metamorphic rocks is strong, influenced by their parent rock and the alteration processes that the rock was subjected [19]. *κ* and NRM increase by an order of quartzite, marble, and gneiss. *Q* values of marble and gneiss are lower than 1, suggesting the dominant contribution of induced magnetization, whereas quartzite has *Q* values >1, indicating a strong contribution of remanent magnetization (Table 4).

**Figure 6:**
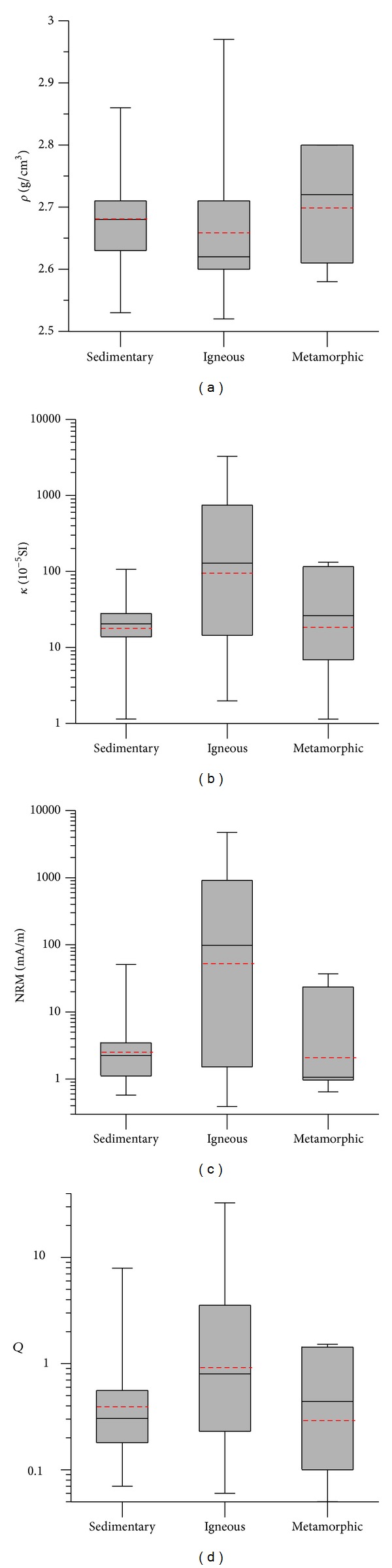
Box plot for (a) *ρ*, (b) *κ*, (c) NRM, and (d) *Q* of sedimentary, igneous, and metamorphic rocks. Red dash lines indicate the average values of the corresponding petrophysical parameters.

In short, igneous rock has the lowest average *ρ* value but the highest NRM, *κ*, and *Q* values, followed by those of sedimentary rock, and metamorphic rock has the highest average *ρ* value, with the lowest NRM and *κ* (Table 5 and Figure 6). Igneous rock has wide distribution and high discretion of *κ* and NRM; their values are over 3 to 5 orders of magnitude (Table 5 and Figure 6).

## 5. Implications for Magnetic Anomalies along the Profile

**Figure 7:**
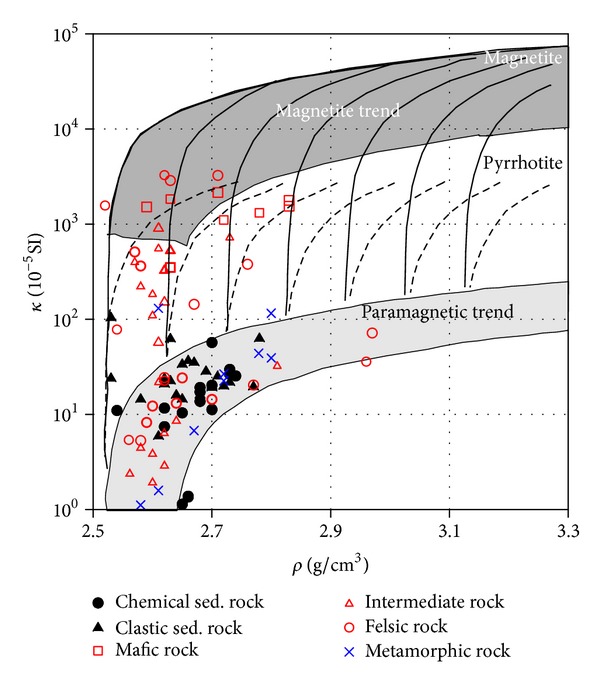
Bivariate distribution of density (*ρ*) versus magnetic susceptibility (*κ*) for all samples sorted for major lithologies.

**Figure 8:**
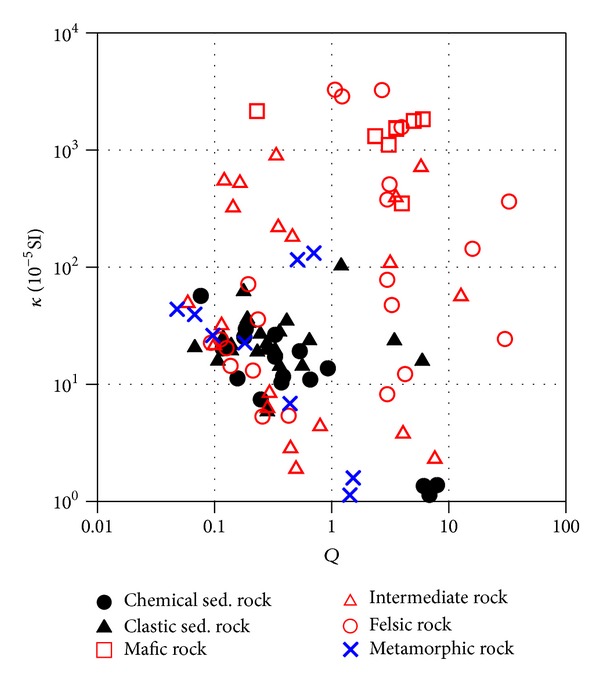
Bivariate distribution of magnetic susceptibility (*κ*) and Köenigsberger ratio (*Q*) for all samples sorted for major lithologies.

It has been found that magnetic susceptibility-density (*κ*-*ρ*) and magnetic susceptibility-*Q* value (*κ*-*Q*) plots are especially useful for analyzing and using petrophysical data; in particular, the *κ*-*ρ* diagram has now been modified with empirically derived typical trend lines describing some of the general features observed in the large sample collection [2]. Bivariate plots of *κ*, *ρ*, and *Q* value distributions for all samples sorted for major lithologies are shown in Figures 7 and 8, respectively. Sedimentary and metamorphic rocks are generally denser and lie in the paramagnetic susceptibility field and in the low *Q* region. Felsic granite usually has low *ρ* with weak magnetization, dominated by paramagnetism. Intermediate rocks show intermediate *κ* and *ρ*, often with a dominant paramagnetic component. In contrast, mafic rocks usually present high *κ* and remanence (high *Q* values), which is associated with the occurrence of ferrimagnetic magnetite, which may cause high variable magnetic anomalies when the strike and dip varies. *κ* and NRM are normally dominated by one of the basic magnetic mineral property types, the difference between these three types being several orders of magnitude.

**Figure 9:**
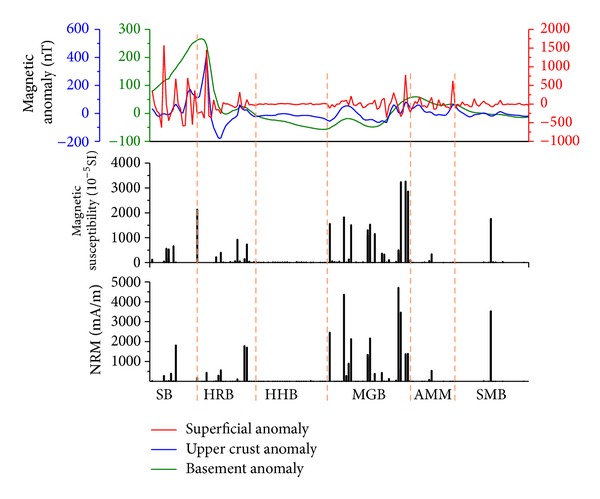
Comparison between different components of lithospheric magnetic anomaly and the NRM and magnetic susceptibility of outcropped rock samples along the profile. Data of the lithospheric magnetic anomalies are compiled from Yuan et al. [14].

Yuan et al. [14] and Wang et al. [20] obtained three components of the lithospheric magnetic field along the Suhbaatar-Ulaanbaatar-Dalandzadgad profile by processing the magnetic survey data. Three components of the lithospheric magnetic anomalies (basement, upper crust, and superficial anomalies) and magnetic parameters of rock samples outcropped along the profile are compared in Figure 9. In Selenge belt (SB), Haraa belt (HRB), and Middle Gobi belt (MGB) areas where igneous rock is widely present, *κ* and NRM are variable (Table 6), magnetic anomalies change dramatically. Hangay-Hentey belt (HHB), Argun-Mongolian Massif (AMM), and Southern Mongolian belt (SMB) areas, where are dominated by sedimentary rock with relatively constant (i.e., small deviations) and weak magnetization (Table 6), show much flatter magnetic anomalies (Figure 9). As previously discussed, igneous rock generally has strong magnetization and considerable variations in NRM and *κ*, whereas magnetization of sedimentary rock is much weaker. Their presence in different areas and depth could be one of the most possible reasons for the different lithospheric magnetic anomaly pattern along the profile.

## 6. Conclusions

From the large amount of petrophysical data of rock samples from the Suhbaatar-Ulaanbaatar-Dalandzadgad geophysical profile, the following important general conclusions describing the variation in density, magnetic susceptibility, and NRM can be drawn.

1. The lower limit of density for all rocks is 2.53 g/cm^3^ and the upper limit is 2.97 g/cm^3^. Such density contrast would result in variable gravity anomalies along the profile.
2. Magnetic susceptibility of sedimentary rocks, metamorphic rocks, and part of felsic to intermediate rocks follows the paramagnetic trend, whereas that of mafic rocks follows the magnetite trend. Magnetic susceptibility and NRM are variable, covering 5-6 orders of magnitude. It makes a variable induced magnetization and further links to complex magnetic anomalies in ground surface.
3. The contribution of remanent magnetization to the production of magnetic anomalies is generally small among sedimentary and metamorphic rocks and part rocks of felsic to intermediate composition, as indicated by the low *Q* values (ratio of remanent to induced magnetization); in contrast, rocks of intermediate to mafic composition have a dominant remanent magnetization as indicated by high *Q* values, and magnetic anomalies produced by these rocks may be variable. The presence of strong remanent magnetization that may alter the direction of the total magnetization would complicate the inversion of magnetic data. Consequently, more attention should be paid to the direction of total magnetization during the inversion of magnetic data.
4. Spatial distribution of rocks with different lithologies controls the pattern of lithospheric magnetic anomaly along the profile.

## Figures and Tables

**Table 1:** Statistical results of density (*ρ*), magnetic susceptibility (*κ*), natural remanent magnetization (NRM), and Köenigsberger ratio (*Q*) of different sedimentary rocks from the Suhbaatar-Ulaanbaatar-Dalandzadgad profile in Mongolia.

Lithology		*ρ* (g/cm^3^)	NRM (mA/m)	*κ* (10^−5^ SI)	*Q *
	Dolostone (*n* = 10)	2.68~2.73	3.5~4.1	19.2~26.5	0.33~0.53
		2.71 ± 0.04	3.8 ± 0.4	22.6 ± 5.2	0.4 ± 0.14
Chemical sedimentary rocks	Limestone (*n* = 40)	2.66~2.86	0.7~5.1	1.4~25.4	0.12~7.93
		2.72 ± 0.06	1.5 ± 1.7	10.2 ± 9.2	0.5 ± 3.41
	Siliceous rocks (*n* = 55)	2.54~2.82	0.6~3.3	1.1~56.9	0.08~6.84
		2.66 ± 0.07	1.7 ± 0.9	9.1 ± 17.1	0.55 ± 2.72
All chemical sedimentary rocks		2.54~2.86 2.69 ± 0.07	0.6~5.1 1.7 ± 1.3	1.1~56.9 10.6 ± 13.9	0.08~7.93 0.51 ± 2.70

	Mudstone (*n* = 20)	2.62~2.77	0.6~3.3	14.8~25.9	0.07~0.56
		2.69 ± 0.07	1.3 ± 1.2	20.2 ± 4.5	0.16 ± 0.23
Clastic rocks	Sandstone (*n* = 75)	2.58~2.78	0.7~5.9	6.1~64.4	0.11~0.41
		2.68 ± 0.06	2.2 ± 1.6	24.8 ± 17.5	0.25 ± 0.09
	Siltstone (*n* = 35)	2.53~2.70	1.8~51.1	16.4~106.5	0.19~5.93
		2.61 ± 0.06	11.3 ± 21.3	30.3 ± 34.2	0.94 ± 2.3
All clastic rocks		2.53~2.78 2.66 ± 0.06	0.6~51.1 3.1 ± 13.9	6.1~106.5 25.2 ± 21.7	0.07~5.93 0.33 ± 1.36

*Note*. The statistical results of different parameters are given as minimum~maximum, mean value ± standard deviation. For *ρ*, the arithmetic mean is used, and for others, the geometric means are given.

**Table 2:** Statistical results of *ρ*, *κ*, NRM, and *Q* values of different igneous rocks.

Lithology		*ρ* (g/cm^3^)	NRM (mA/m)	*κ* (10^−5^ SI)	*Q *
Extrusive rocks	Basalt (*n* = 45)	2.59~2.83	196.5~4378.0	350.3~2150.4	0.23~6.00
		2.72 ± 0.09	1379.8 ± 1435.3	1303.5 ± 547.7	2.66 ± 1.73
	Dacite (*n* = 30)	2.58~2.71	1.1~4726.5	12.2~3275.7	0.21~32.69
		2.63 ± 0.04	300.2 ± 1902.5	348.3 ± 1656	2.17 ± 12.65
All extrusive rocks		2.58~2.83 2.69 ± 0.09	1.1~4726.5 717.7 ± 1582.6	12.2~3275.7 740.4 ± 1106.8	0.21~32.69 2.44 ± 8.16

Pyroclastic rocks	Tuff (*n* = 45)	2.52~2.77	0.5~2465.3	5.3~1568.3	0.09~30.13
		2.63 ± 0.08	14.2 ± 823.6	41.5 ± 510.8	0.86 ± 10.40

Hypabyssal rocks	Porphyry (*n* = 25)	2.56~2.60	0.4~632.4	2.0~509.9	0.43~7.59
		2.57 ± 0.02	10.0 ± 276.2	14.4 ± 222.5	1.75 ± 2.92

	Diorite (*n* = 15)	2.85~2.97	3.3~5.5	35.9~71.7	0.19~0.23
		2.93 ± 0.07	4.3 ± 1.5	50.7 ± 25.3	0.21 ± 0.03
Plutonic rocks (*n* = 160)	Syenite (*n* = 10)	2.59~2.76	9.8~449.4	8.2~378.6	2.98~2.98
		2.68 ± 0.12	66.2 ± 310.9	55.8 ± 261.9	2.98 ± 0.0
	Granite (*n* = 135)	2.57~2.81	0.5~1826.5	3.0~933.7	0.06~12.71
		2.64 ± 0.06	38.4 ± 562.4	79.7 ± 292.0	0.49 ± 2.88
All plutonic rocks		2.58~2.83 2.69 ± 0.09	1.1~4726.5 717.7 ± 1582.6	12.2~3275.7 740.4 ± 1106.8	0.21~32.69 2.44 ± 8.16

**Table 3:** Statistical results of *ρ*, *κ*, NRM, and *Q* values of mafic, intermediate, and felsic igneous rocks.

Lithology	*ρ* (g/cm^3^)	NRM (mA/m)	*κ* (10^−5^ SI)	*Q *
Mafic rocks (*n* = 45)	2.59~2.83	196.5~4378.0	350.3~2150.4	0.23~6.00
	2.72 ± 0.09	1379.8 ± 1435.3	1303.5 ± 547.7
Intermediate rocks (*n* = 115)	2.52~2.97	0.5~4726.5	5.3~3275.7	0.09~32.69
	2.67 ± 0.12	37.8 ± 1279.5	79.7 ± 1095.2	1.19 ± 9.21
Felsic rocks (*n* = 145)	2.56~2.81	0.4~1826.5	2.0~933.7	0.06~12.71
	2.63 ± 0.06	30.7 ± 547.5	57.4 ± 287.5	0.55 ± 3.02

**Table 4:** Statistical results of *ρ*, *κ*, NRM, and *Q* values of different metamorphic rocks.

Lithologies	*ρ* (g/cm^3^)	NRM (mA/m)	*κ* (10^−5^ SI)	*Q *
Gneiss (*n* = 25)	2.67~2.80	0.8~37.0	6.9~132.1	0.05~0.70
	2.72 ± 0.08	3.9 ± 16.7	41.3 ± 55.8	0.24 ± 0.28
Marble (*n* = 10)	2.72~2.80	1.1~1.6	22.6~39.6	0.07~0.18
	2.76 ± 0.06	1.3 ± 0.4	29.9 ± 12.0	0.11 ± 0.08
Quartzite (*n* = 10)	2.58~2.61	0.6~1.0	1.1~1.6	1.43~1.52
	2.60 ± 0.02	0.8 ± 0.2	1.3 ± 0.3	1.48 ± 0.07

**Table 5:** Statistical results of *ρ*, *κ*, NRM, and *Q* values of rocks with three major lithologies.

Lithologies	*ρ* (g/cm^3^)	NRM (mA/m)	*κ* (10^−5^ SI)	*Q *
Sedimentary rocks (*n* = 235)	2.53~2.86	0.6~51.1	1.1~106.5	0.07~7.93
	2.68 ± 0.07	2.4 ± 10.3	17.6 ± 19.9	0.39 ± 2.05
Igneous rocks (*n* = 305)	2.42~2.97	0.4~4726.5	2.0~3275.7	0.06~32.69
	2.66 ± 0.10	56.1 ± 1138.4	106.7 ± 860.0	0.94 ± 6.30
Metamorphic rocks (*n* = 45)	2.58~2.80	0.6~37.0	1.1~132.1	0.05~1.52
	2.70 ± 0.09	2.1 ± 13.3	18.0 ± 48.4	0.30 ± 0.57

**Table 6:** Statistical results of *ρ*, *κ*, NRM, and *Q* values of rocks in different tectonic units.

Tectonic units	Major lithologies	*ρ* (g/cm^3^)	NRM (mA/m)	*κ* (10^−5^ SI)	*Q *
SB	Granite, dacite	2.60~2.85	1.1~1826.5	12.2~2150.4	0.11~12.71
		2.67 ± 0.08	58.5 ± 494.5	161.8 ± 648.1	0.47 ± 3.65
HRB	Granite, marble, and sandstone	2.57~2.97	0.5~1790.8	3.0 ~933.7	0.05~5.79
		2.70 ± 0.10	10.6 ± 551.2	50.7 ± 275.1	0.28 ± 1.53
HHB	Sandstone, tuff	2.52~2.77	0.7~38.6	1.1~36.0	0.11~7.59
		2.68 ± 0.05	2.1 ± 7.6	13.3 ± 8.6	0.41 ± 2.48
MGB	Dacite, granite, and basalt	2.52~2.96	0.4~4726.5	2.0~3275.7	0.07~32.69
		2.66 ± 0.09	50.7 ± 1320.0	123.1 ± 989.3	1.17 ± 7.87
AMM	Siliceous rock, limestone	2.53~2.70	0.9~51.1	1.4~106.5	0.43~7.93
		2.60 ± 0.06	3.3 ± 16.1	8.2 ± 35.4	1.09 ± 2.51
SMB	Siliceous rock, clastic rock	2.58~2.73	1.1~33.4	10.4~37.6	0.11~3.43
		2.65 ± 0.05	3.0 ± 11.8	23.1 ± 9.9	0.32 ± 1.21
